# Supplementary material for: Comparative genomic analyses reveal diverse virulence factors and antimicrobial resistance mechanisms in clinical Elizabethkingia meningoseptica strains
Source: PLoS One. 2019 Oct 10;14(10):e0222648. doi: 10.1371/journal.pone.0222648 (PMC6786605; doi:10.1371/journal.pone.0222648)
Supplement: S1 Fig — The selected E. meningoseptica strains are those listed on the NCBI database (cutoff date 11/15/2018): https://www.ncbi.nlm.nih.gov/genome/?term=Elizabethkingia. (DOCX) [file pone.0222648.s001.docx]

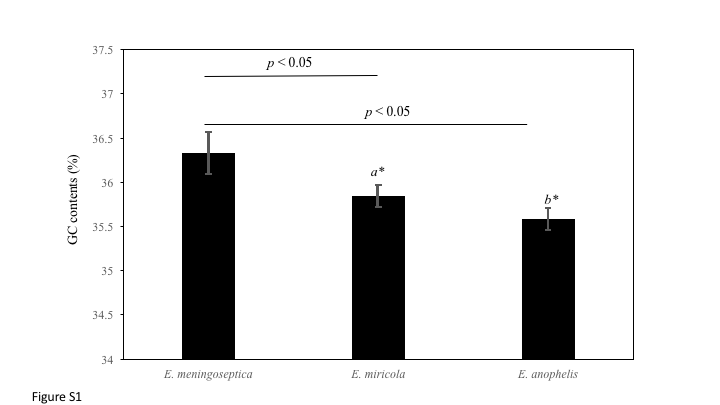


**S1 Fig. Comparisions of the GC contents in *E. meningoseptica, E. anophelis* and *E. miricola***. The selected *E. meningoseptica* strains are those listed on the NCBI database (cutoff date 11/15/2018): <https://www.ncbi.nlm.nih.gov/genome/?term=Elizabethkingia>.
