# Supplementary material for: Comparative genomic analyses reveal diverse virulence factors and antimicrobial resistance mechanisms in clinical Elizabethkingia meningoseptica strains
Source: PLoS One. 2019 Oct 10;14(10):e0222648. doi: 10.1371/journal.pone.0222648 (PMC6786605; doi:10.1371/journal.pone.0222648)
Supplement: S2 Fig — (DOCX) [file pone.0222648.s002.docx]

**
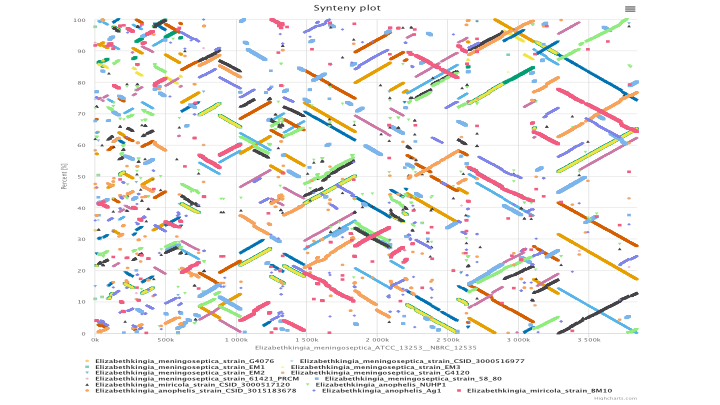
**

**S2 Fig. Synteny plots show the conservation of gene order among the selected *Elizabethkingia* genomes.**
