# Supplementary material for: Comparative genomic analyses reveal diverse virulence factors and antimicrobial resistance mechanisms in clinical Elizabethkingia meningoseptica strains
Source: PLoS One. 2019 Oct 10;14(10):e0222648. doi: 10.1371/journal.pone.0222648 (PMC6786605; doi:10.1371/journal.pone.0222648)
Supplement: S3 Fig — Tree was constructed for 32 genomes with a core of 1170 genes per genome, 37440 in total. The core has 405494 AA-residues/ bp per genome, 12975808 in total. (DOCX) [file pone.0222648.s003.docx]

**
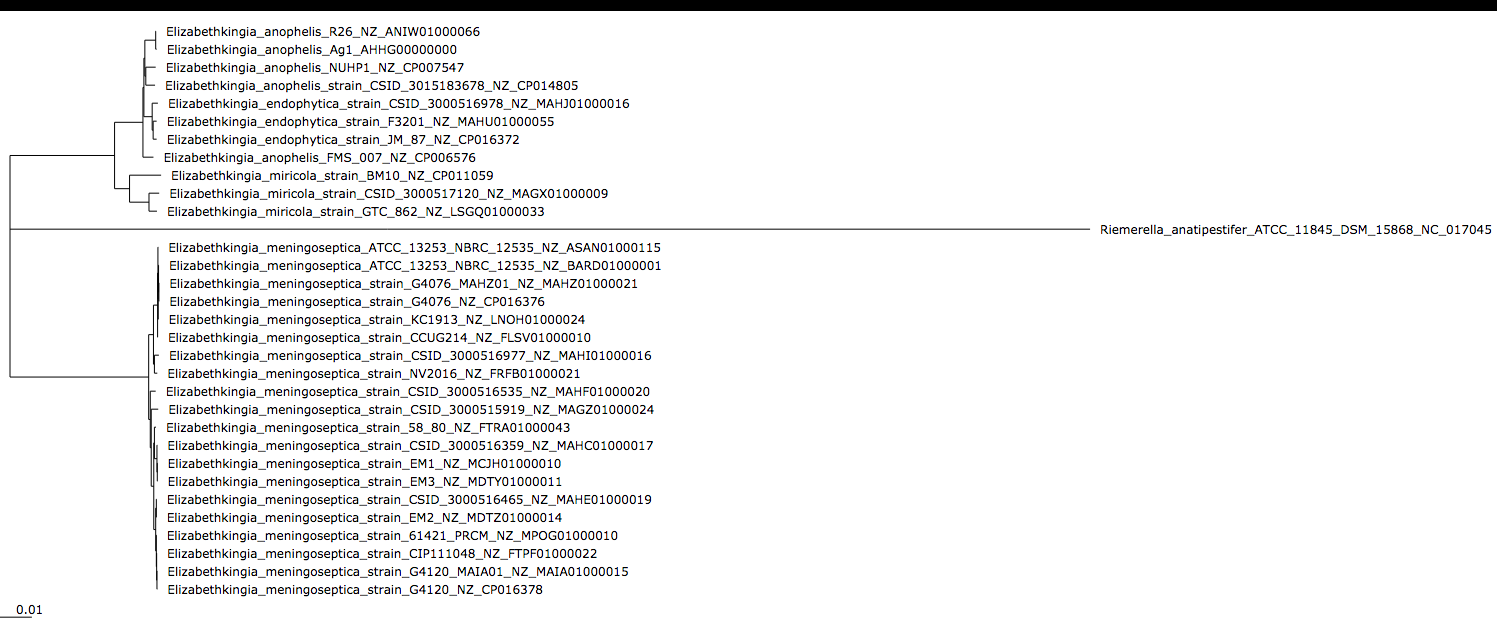
S3 Fig. Phylogenetic relationship among the selected *Elizabethkingia*.** Tree was constructed for 32 genomes with a core of 1170 genes per genome, 37440 in total. The core has 405494 AA-residues/ bp per genome, 12975808 in total.
