# Supplementary material for: Comparative genomic analyses reveal diverse virulence factors and antimicrobial resistance mechanisms in clinical Elizabethkingia meningoseptica strains
Source: PLoS One. 2019 Oct 10;14(10):e0222648. doi: 10.1371/journal.pone.0222648 (PMC6786605; doi:10.1371/journal.pone.0222648)
Supplement: S4 Fig — EDGAR was used for Venn diagrams. (DOCX) [file pone.0222648.s004.docx]

**
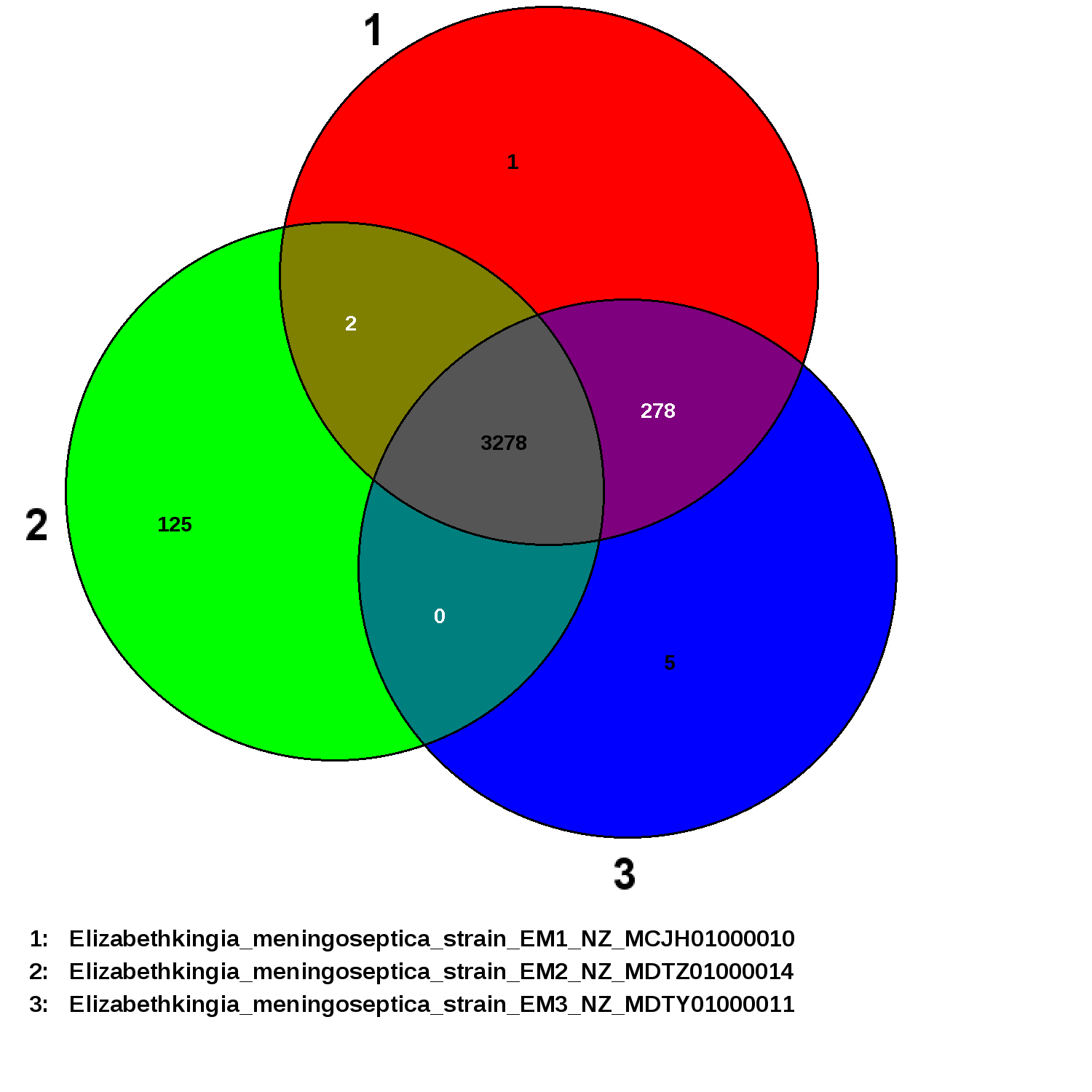
**

**S4 Fig. The shared genes among the selected *E. meningoseptica* Em1, Em2 and Em3.** EDGAR was used for Venn diagrams.
