# Supplementary material for: Comparative genomic analyses reveal diverse virulence factors and antimicrobial resistance mechanisms in clinical Elizabethkingia meningoseptica strains
Source: PLoS One. 2019 Oct 10;14(10):e0222648. doi: 10.1371/journal.pone.0222648 (PMC6786605; doi:10.1371/journal.pone.0222648)
Supplement: S5 Fig — The cells were cultured by shaking in TSB at 37°C to obtained the initial inocula and the cell density was adjusted to the same OD at 600 nm (0.1). 200 μl were inoculated on 96-well plates for at least 24 hours. The biofilm assay was carried out using crystal blue staining. (DOCX) [file pone.0222648.s005.docx]

**S5 Fig. *In vitro* biofilm assay in the selected *E. meningoseptica*.** The cells were cultured by shaking in TSB at 37 °C to obtained the initial inocula and the cell density was adjusted to the same OD at 600 nm (0.1). 200 μl were inoculated on 96-well plates for at least 24 hours. The biofilm assay was carried out using crystal blue staining.
